# A pediatric case developing critical abdominal distension caused by a combination of humidified high-flow nasal cannula oxygen therapy and nasal airway

**DOI:** 10.1186/s40981-017-0143-0

**Published:** 2018-01-05

**Authors:** Satoki Inoue, Yumiko Tamaki, Shota Sonobe, Junji Egawa, Masahiko Kawaguchi

**Affiliations:** Department of Anesthesiology and Division of Intensive Care, Nara Medical University, 840 Shijo-cho Kashihara, Nara, 634-8522 Japan

**Keywords:** Humidified, High-flow nasal cannula, Abdominal distention, Nasal airway, Complication, Pediatric patient

## Abstract

**Background:**

We describe a pediatric patient who suffered from critical abdominal distention caused by a combination of humidified, high-flow nasal cannula (HHFNC) oxygen therapy and nasal airway.

**Case presentation:**

A 21-month-old boy with a history of chronic lung disease was admitted to the intensive care unit (ICU). Immediately after admission, his airway was established using a tracheal tube and mechanical ventilation was started. Five days after the commencement of mechanical ventilation, finally, his trachea was extubated. Immediately after extubation, HHFNC therapy at 20 L/min with an FiO_2_ of 0.35 was applied. However, severe stridor was observed, then a nasal airway was placed in the left nostril. However, he became restless. Critical abdominal distention was observed. A subsequent chest X-ray revealed that the nasal airway was placed too deeply, and the gastrointestinal air was severely accumulated. Immediately, the nasal airway was removed, and HHFNC flow was reduced to 10 L/min. Frequent suctioning and continuous gastric drainage were required, which achieved gradual improvement of respiratory condition.

**Conclusions:**

We need to recognize that HHFNC therapy is one of the positive pressure ventilation system. Therefore, HHFNC therapy might cause the similar adverse events to noninvasive pressure ventilation.

## Background

Humidified, high-flow nasal cannula (HHFNC) oxygen therapy has allowed optimal humidification of inspired gas at high flows, which could create a distending pressure-like nasal continuous positive airway pressure (CPAP) [1]. There is a small but growing body of information from clinical trials that support the use of HHFNC as an alternative oxygen interface for critically ill patients across the entire age spectrum, from premature neonates to adults [2–4]. In addition, it has been reported that HHFNC is equivalent to more traditional non-invasive ventilation support, such as continuous or bi-level positive airway pressure (CPAP or BiPAP) [3, 4]. Therefore, HHFNC has become a popular and easy respiratory option for pediatric patients despite the lack of clear practical guidance for safety operation. We describe a pediatric patient who suffered from critical abdominal distention caused by a combination HHFNC therapy and nasal airway.

## Case presentation

**Fig. 1:**
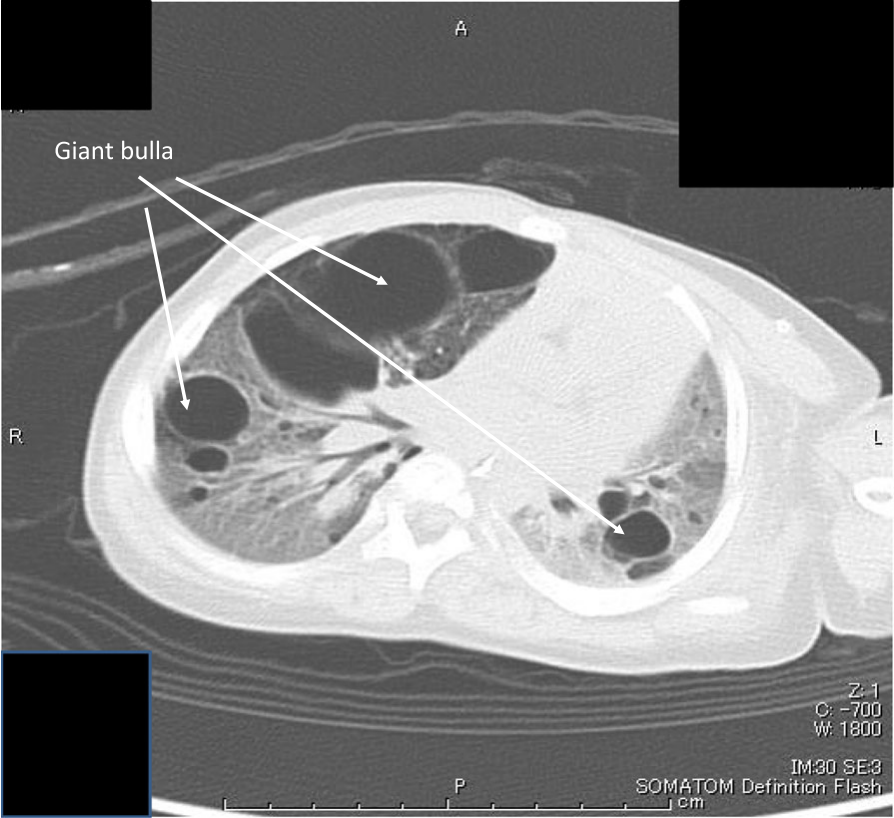
A chest CT at healthy status. Several giant bullae in the bilateral lungs were observed

The consent of patient’s next of kin was obtained; however, institutional review board approval was exempted because neither the ethical problem nor the description to identify the patient was included in this case report. A 21-month-old boy with acute or chronic lung disease was admitted to the intensive care unit (ICU). He was 63 cm in height and weighed 7.9 kg. He was bone on the 24^th^ week of gestation and weighed 645 g and had been provided with home oxygen therapy with 3 L/min oxygen administration. His chest computed tomography showed several giant bullae in the bilateral lungs (Fig. 1). He had gastroesophageal reflux and an intestinal feeding tube (6.5 F) and a gastric drainage tube (6 F) indwelled.

He had been suffering from upper respiratory infection and fever for a couple of days. He gradually developed hypoxia (SpO_2_ < 90%) with oxygen administration. Immediately after admission, his airway was established using a cuffed 3.5-mm tracheal tube. Immediately after the beginning of mechanical ventilation, 10-cm H_2_O positive end-expiratory pressure (PEEP) combined with moderate pressure support (8 cm H_2_O) was applied.

His respiratory status gradually improved. Five days after the commencement of mechanical ventilation, finally, his trachea was extubated. Immediately after extubation, HHFNC therapy at 20 L/min with an FiO_2_ of 0.35 (Optiflow system™, MR850 heated humidified RT202 delivery tubing; Fisher and Paykel Healthcare Ltd., Auckland, New Zealand) was applied through nasal cannula (Optiflow™ Junior Nasal Cannula Infant; Fisher & Paykel, Auckland, New Zealand).

However, severe stridor because of upper airway obstruction was observed, then a 10-cm-long 3.5-mm hand-made nasal airway (Portex™, Smith Medical, Kent, UK) was placed in the left nostril (10 cm depth). It was not confirmed whether or not inserting the tube too deeply because he violently resisted being checked with a laryngoscopy. But, stridor or retractive breathing was not observed. Therefore, HHFNC therapy was restarted with this airway position.

**Fig. 2:**
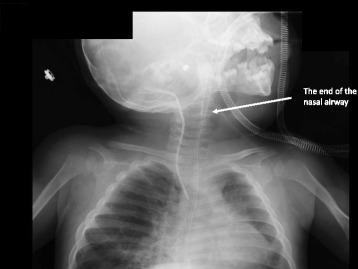
A cervical X-ray at deteriorated respiratory status. A subsequent cervical X-ray revealed that the nasal airway was placed too deeply

**Fig. 3:**
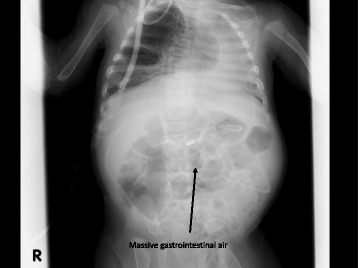
A chest-abdominal X-ray at deteriorated respiratory status. A subsequent chest-abdominal X-ray revealed that gastrointestinal air was severely accumulated

HHFNC was conducted smoothly for a while; however, he became restless and then started agonizing heavily with SpO_2_ reduction down to 70%. Critical abdominal distention was observed, and forced gastric drainage was started through a gastric tube. However, the critical situation was not improved. A subsequent chest X-ray revealed that the nasal airway was placed too deeply (Fig. 2), and the gastrointestinal air was severely accumulated (Fig. 3).

Immediately, the nasal airway was removed and HHFNC flow was reduced to 10 L/min. Frequent suctioning and continuous gastric drainage were required, which achieved gradual improvement of respiratory condition. Finally, HHFNC therapy was successfully terminated.

## Conclusions

It is supposed that the deeply inserted nasal airway caused critical abdominal distention by insufflating the HHFNC flow into the gastrointestinal rather than the respiratory tract. In the early stage before the development of critical abdominal distention, the HHFNC flow might have partially supported his breathing; however, it is likely that the HHFNC flow gradually became ineffective for supporting his breathing because of aerophagia. In addition, his gastroesophageal reflux might have worsened the situation because aerophagia is possibly accelerated by noninvasive positive airway pressure in patients with gastroesophageal reflux [5]. Abdominal compartment syndrome related to noninvasive ventilation was also reported [6]. In a pediatric model, a linear relationship between flow and pressures measured in the pharynx (pressure = − 0.375 + 0.138 × flow) with the closed-mouth condition was reported. According to this model, 2.4 cm H_2_O of positive pressure was generated in our patient at that time. This low level of pressure might unusually force air into the stomach; however, the positive pressure drastically increased with a high nasal prong-to-nares ratio and mouth closed status [7]. Therefore, it might be a little wonder that a positive pressure high enough to open the esophageal sphincter was generated according to the circumstances, which caused the gastrointestinal overdistention. To confirm the location of the tip of the nasal airway, we could have used a fine fiberoptic bronchoscopy as an option. We should have carefully monitored the patient considering the development of this adverse event.

In conclusion, we need to recognize that HHFNC therapy is one of the positive pressure ventilation system. Therefore, HHFNC therapy might cause the similar adverse events to noninvasive pressure ventilation. This time, we would like to underline that critical abdominal overdistention could happen by HHFNC therapy.

## Abbreviations

BiPAP: Bi-level positive airway pressure
CPAP: Continuous positive airway pressure
HHFNC: Humidified, high-flow nasal cannula
ICU: Intensive care unit
PEEP: Positive end-expiratory pressure
